# Flapless and Conjunctiva-Sparing Technique for Transscleral Fixation of Intraocular Lens to Correct Refractive Errors in Eyes without Adequate Capsular Support

**DOI:** 10.1155/2023/4032011

**Published:** 2023-04-19

**Authors:** Wei Lou, Ziang Chen, Yang Huang, Haiying Jin

**Affiliations:** ^1^Department of Ophthalmology, Shanghai East Hospital, Tongji University School of Medicine, Shanghai, China; ^2^Department of Ophthalmology, Shanghai Tenth People's Hospital, Tongji University School of Medicine, Shanghai, China

## Abstract

**Purpose:**

To evaluate refractive outcomes, intraocular lens (IOL) power calculation, and IOL position following a novel conjunctiva-sparing transscleral fixation technique.

**Methods:**

Forty-one eyes of 40 patients managed with a flapless transscleral-sutured technique were included. Preoperative and postoperative refractive errors (spherical equivalents, SE) were compared. IOL position was assessed on the Scheimpflug images. IOL power was calculated by SRK/T, Holladay 1, and Hoffer Q formulas.

**Results:**

The mean age was 57.39 ± 14.83 years (range: 26 to 79 years), and the mean follow-up was 7.46 ± 6.42 months (range: 1 to 24 months). Surgical indications were aphakia (*n* = 14), subluxated lenses (*n* = 3), and IOL dislocation (*n* = 24). The SE was 4.50 ± 6.38 diopter (D) (range: −3.75 to 13.75 D) preoperatively and −1.68 ± 1.57 D (range: −5.50 to 1.13 D) postoperatively (*P* < 0.001). The mean tilt angle and decentration were 2.90° ± 1.93° (range: 0.39° to 9.10°) and 0.23 ± 0.19 mm (range: 0.02 to 0.94 mm) vertically, and 1.75° ± 1.41° (range: 0.24° to 7.65°) and 0.18 ± 0.19 mm (range: 0.02 to 1.06 mm) horizontally, which were clinically insignificant. All three IOL formulas produced myopic errors (range: −0.29 to −0.50 D). The SRK/T had the lowest median absolute error (0.55 D), followed by the Holladay 1 (0.70 D) and the Hoffer Q (0.74 D). The three formulas had the same percentage of prediction errors (PEs) within ±0.5 D (43.48%), while the Hoffer Q had the highest percentage of PEs within ±1.0 D (82.61%).

**Conclusion:**

The present technique can serve as an alternative approach for transscleral IOL fixation and refractive correction in eyes with compromised capsular support, ensuring the stability of IOLs and reasonable IOL power calculation accuracy.

## 1. Introduction

Cataract surgery has evolved from the restoration of visual function to refractive correction. Refractive correction in eyes with inadequate capsular support can be accomplished using anterior chamber IOL, iris-fixated IOL, or scleral-fixated IOL [1–5]. Implanting a posterior chamber IOL via a scleral-fixated method has several inherent advantages over other techniques, allowing the implanted IOL to be positioned closer to the original crystalline lens with a reasonable distance from anterior segment structures. Transscleral-sutured fixation, a commonly performed scleral-fixated technique, typically requires the large opening of conjunctival tissues and the creation of scleral flaps to bury the suture ends and knots [6–8]. The most commonly used 10-0 polypropylene in sutured-fixation methods is prone to loosening and breakage, leading to IOL tilt, decentration, or even up to a dislocation rate of 18% to 28%, and thus the occurrence of significant postoperative refractive errors, of which IOL tilt angle larger than 15° cannot be corrected with spectacles [9, 10]. We have previously described a flapless and conjunctiva-sparing technique for transscleral IOL fixation using the 8-0 polypropylene [5]. We herein present the results of refractive correction and IOL position after this minimally invasive surgical technique.

## 2. Methods

The study was approved by the local ethics committee, and the principles of the Declaration of Helsinki were strictly followed throughout the study. The surgical indications were 1) aphakia, 2) subluxated lenses with more than 8-clock-zonulodialysis that had less chance of preserving the capsular bag, and 3) dislocated IOLs. Before surgery, surgical procedures and potential risks were explicitly explained; meanwhile, informed consent was acquired from all patients.

A standardized ophthalmological examination was conducted at preoperative and postoperative visits, including slit-lamp examination, uncorrected visual acuity (UCVA), best corrected visual acuity (BCVA), noncontact intraocular pressure (IOP), manifest refraction, ultrawidefield fundus image (Optos, Optos PLC, Dunfermline, United Kingdom), and foveal scans (HD-5000, Carl Zeiss AG, Oberkochen, Germany). Keratometry, anterior chamber depth (ACD), axial length (AL), and other ocular biometry were measured using IOLMaster 700 (Carl Zeiss AG, Oberkochen, Germany) to determine implanted IOL power preoperatively. Anterior segment images were captured by the Pentacam (Oculus Optikgeräte GmbH, Wetzlar, Germany) at postoperative follow-up time points to assess IOL position (IOL tilt angle and decentration).

### 2.1. Surgical Techniques

**Figure 1:**
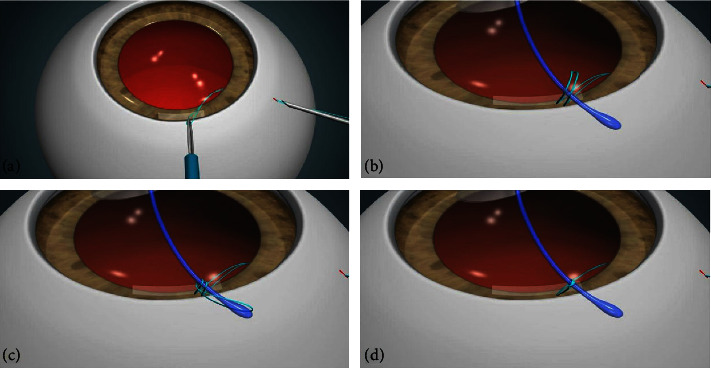
Schematic figures of performing the modified cow-hitch knot to fixate the intraocular lens (IOL): (a) the suture-in-needle technique was performed to introduce the suture into the eye from the fixation site, and the loop was externalized by forceps. (b) The loop of suture was passed proximally towards the optic of the IOL, then behind the twirl, and moved distally to lasso the end of the haptic. (c) The suture was looped around the end of the haptic. (d) The modified cow-hitch was finally tightened.

The surgeries were performed under retrobulbar anesthesia by one of the authors (J. H). The supplemental video (see Videos 1 and 2, Supplement Digital Content 1 and 2) demonstrates the procedures. A twin-armed single 8-0 polypropylene suture (Prolene, Polypropylene Suture; Ethicon, Johnson & Johnson, New Brunswick, New Jersey) was cut at its middle. The suture was introduced into the eye using a 30-gauge needle from the fixation site, 2.0 mm posterior to the limbus, with the aid of a suture-in-needle technique [11]. The suture loop inside the globe was grasped and externalized from the eye by an end-open forceps, through the clear corneal incision. The double-strand suture was twined around the haptic on the centripetal side. Then, the loop was lassoed around the end of the haptic. The suture was further pulled to fasten the modified cow-hitch knot (Figure 1). The same set of manipulations was performed to fixate the opposite haptic. For cases with subluxated lenses, the present technique was performed after phacoemulsification by the aid of temporary capsule retractors. A limbal vitrectomy was conducted if necessary. For cases with a dislocated IOL, the haptics were externalized through corneal paracentesis before the knotting technique was adopted. After introducing both haptics into the eye and adjusting the suture tensions to center the IOL, the conjunctiva-sparing and flapless fixation technique described in our previous publication was adopted to fixate the suture to the scleral wall [5]. In short, performing the back-and-forth intrascleral suture pass for definitive fixation. The two ends of the suture were knotted into the sclerotomy. Another overhand knot 2.0  to 3.0 mm from the fixation knot was anchored intrasclerally by the aid of a 30-gauge needle.

### 2.2. IOL Tilt and Decentration Measurement

**Figure 2:**
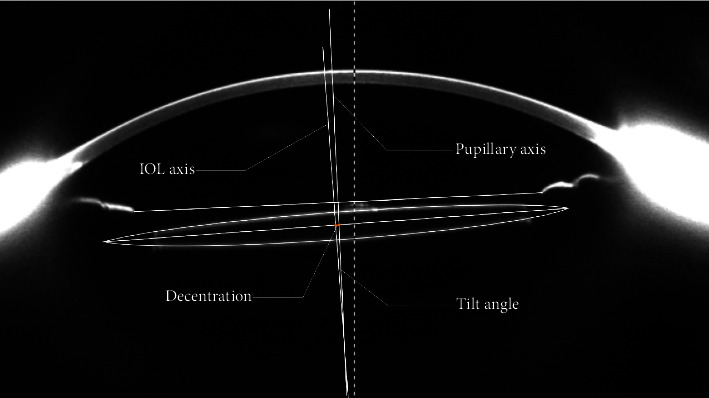
Scheimpflug analysis of intraocular lens (IOL) position. The tilt angle was defined as the angle between two axes. The decentration was calculated as the distance between the IOL axis and the pupillary axis.

The Pentacam examination was performed by one experienced technician on all patients under scotopic conditions. Two Pentacam Scheimpflug images on 90° and 180° meridians were analyzed using ImageJ software (version 1.8.0) to measure postoperative IOL position (IOL tilt and decentration). The anterior and posterior IOL surfaces were first plotted to determine the IOL axis, the line passing the IOL midpoint and perpendicular to the line of intersection of the IOL surfaces. The pupillary axis was defined as the line passing the pupil center and connecting the anterior corneal center of curvature, the IOL tilt angle as the angle between the IOL axis and the pupillary axis, and the IOL decentration as the distance between the two axes (Figure 2).

### 2.3. Refractive Prediction Error

As IOL formulas using ACD as variables to calculate IOL power were impractical for aphakia, three formulas (SRK/T, Holladay 1, and Hoffer Q formulas), independent of preoperative ACD, were used. The User Group for Laser Interference Biometry website (https://ocusoft.de/ulib/index.htm) was browsed to determine the optimized lens constant values for each formula. The three formulas were calculated online (https://www.eyecalcs.com). Prediction error (PE) was calculated as the postoperatively measured refractive spherical equivalent (SE) minus the predictive SE of the implanted IOL; the negative PE indicated that the formula produced myopic errors and the positive PE hyperopic errors. The arithmetic PE, the mean absolute error (MAE), the median absolute error (MedAE), and the percentage of eyes with a PE within ±0.50 diopters (D), ±1.00 D, and ±2.00 D were evaluated.

### 2.4. Statistical Analysis

The statistical analyses were executed using SPSS software (version 26; IBM Corp, New York, NY), Prism (version 8.0.1, GraphPad Software, San Diego, CA), and Excel spreadsheet (Microsoft Corp). The normality of data distribution was determined using the Kolmogorov–Smirnov test. The preoperative and postoperative parameters were compared by the paired *t*-test or Wilcoxon signed-rank test based on the data distribution. The Friedman test with Dunn's post-test was applied to compare the arithmetic PEs and AEs between the formulas. The Cochran Q test was utilized to assess the percentage of eyes with PEs within *±*0.50 D and *±*1.00 D. For multiple comparisons, the Bonferroni adjustment was used. Statistical significance was defined as a *P* value less than 0.05. Clinically significant tilt and decentration were defined as tilt angles larger than 7° and decentration larger than 0.4 mm, respectively [12].

## 3. Results

A total of 41 eyes from 40 patients, aged 57.39 ± 14.83 years (range: 26 to 79 years), were included in the study. Twenty-three eyes received IOL implantation, and the other 18 cases with dislocated IOLs received IOL rescue procedures. The surgical indications were aphakia after lens extraction and vitrectomy (*n* = 14), subluxated lens (*n* = 3), and IOL dislocation (*n* = 24) (Table 1). The mean follow-up period was 7.46 ± 6.42 months (range: 1 to 24 months). The IOL types included the ZA9003 (Abbott Medical Optics, Inc), the PY-60AD (HOYA, Corp), and the AR40e (Abbott Medical Optics Inc).

UCVA (logarithm of the minimum angle of resolution (LogMAR)) was improved from 1.28 ± 0.74 LogMAR preoperatively to 0.58 ± 0.40 LogMAR postoperatively (*P* < 0.001). BCVA was improved from 0.52 ± 0.62 LogMAR preoperatively to 0.31 ± 0.36 LogMAR postoperatively (*P* = 0.01) (Table 2). There was no significant difference between the preoperative and postoperative IOP (*P* = 0.82), which was 14.74 ± 3.59 mmHg preoperatively and 14.40 ± 4.95 mmHg postoperatively. The refractive error was 4.50 ± 6.38 D (range: −3.75 to 13.75 D) preoperatively and −1.68 ± 1.57 D (range: −5.50 to 1.13 D) (*P* < 0.001) postoperatively (Table 2).

The mean vertical and horizontal IOL tilt angles were 2.90° ± 1.93° (range: 0.39° to 9.10°) and 1.75° ± 1.41° (range: 0.24° to 7.65°), respectively. The mean amounts of IOL decentration were 0.23 ± 0.19 mm (range: 0.02  to 0.94) vertically and 0.18 ± 0.19 mm (range: 0.02  to 1.06) horizontally (Table 3).

**Figure 3:**
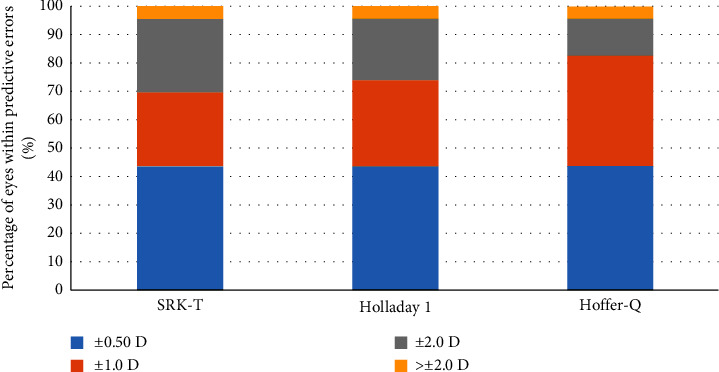
Stacked histogram comparing percentage of eyes within the certain refractive prediction errors of the 3 formulas.

The accuracy of IOL power calculation was assessed in 23 eyes that underwent IOL implantation. All three IOL formulas produced myopic PEs, and the SRK/T formula had a more significant myopic error than the Holladay 1 formula (SRK/T: −0.50 ± 0.97 D vs. Holladay 1: −0.36 ± 0.97 D, *P* = 0.04) and the Hoffer Q formula (SRK/T: −0.50 ± 0.97 D vs. Hoffer Q: −0.29 ± 0.93 D, *P* < 0.001). The mean MAEs of SRK/T, Holladay 1, and Hoffer Q formulas were 0.81 ± 0.72 D, 0.76 ± 0.68 D, and 0.70 ± 0.66 D, respectively. The SRK/T formula had the lowest MedAE (0.55 D), which was statistically lower than the Hoffer Q formula (0.74 D, *P* = 0.02) (Table 4). The percentage of PEs within ±0.5 D was the same for the three formulas (43.48%). The Hoffer Q formula had the highest percentage of PEs within ±1.0 D (82.61%) than the other two formulas (SRK/T: 69.57%; Holladay 1 : 73.91%) (Figure 3). However, there was no statistical significance among the three formulas concerning the percentage of PEs within ±0.5 D, ±1.0 D, and ±2.0 D (all *P* > 0.05).

**Figure 4:**
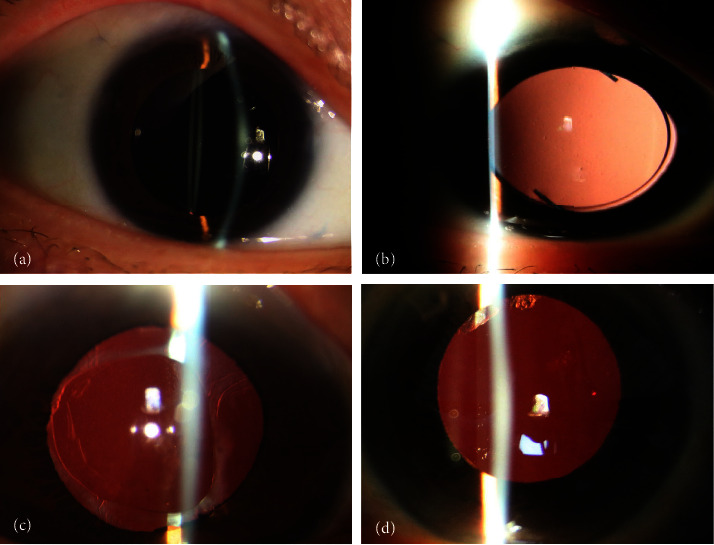
Postoperative pictures of cases receiving the present transscleral fixation: (a, b) twenty-two-month postoperative overview of a case with dislocated IOL. Suture erosion or scleral atrophy was not observed. (c, d) The IOLs were well centered for one case after a three-month follow-up (c) and the other after an eleven-month follow-up (d).

At the time of ciliary sulcus penetration, a mild and temporary hemorrhage was noted in two eyes. There were no other observed intraoperative complications. Postoperatively, hypotony occurred in two eyes. With close observation and routine medications, the IOP normalized within the first week. Transient IOP spike was detected in six eyes, of which five eyes were managed with antihypertensive medications, and the IOP returned to normal after one week. The other one with elevated postoperative IOP, diagnosed with traumatic angle recession glaucoma before surgery, received the implantation of a glaucoma drainage device two months later. During the follow-up period, two cases of IOL pupillary capture and one case of suture exposure were observed, which were managed by paired suture. The IOLs remained well centered (Figure 4). No scleral atrophy, suture lack, chronic corneal edema, hyphema, vitreous hemorrhage, retinal tear, retinal detachment, or IOL redislocation was detected.

## 4. Discussion

Refractive correction in eyes with insufficient capsular support remains challenging. First, current formulas assume the accomplishment of in-the-bag IOL implantation; however, due to different IOL types and surgical techniques, the position of IOLs placed in such eyes can be unpredictable. Second, the accuracy of IOL power calculation would be compromised by factors contributing to ocular biometry measurement errors, including poor visual acuity for eye fixations, comorbidities, and altered refractive index following vitrectomy [13–15]. Moreover, surgeons typically use anterior chamber IOL, iris-fixated IOL, and scleral-fixated IOL to correct refractive errors in the absence of adequate capsular support, all of which are more technically difficult than routine cataract surgery [1–5]. Currently, the flanged fixation technique for three-piece IOLs is a reliable and widely used technique; however, it has limitations on the types of IOLs that can be used. The flanged technique is only practicable in certain types of three-piece IOLs [16, 17]. As a flange created by thermos-cauterization is the key point of the technique, certain types of IOLs, whose haptics are made from materials that cannot be reshaped by thermos-plasticity, are impractical for this technique [16, 17]. Transscleral-sutured fixation remains a vital method to fixate IOLs; compared with anterior chamber IOL and iris-fixated IOL, it places an IOL closer to the original crystalline lens and has the advantages of lower demand for corneal endothelium, iris structure, and angle status [4, 6]. Furthermore, compared with the flanged method, transscleral-sutured fixation is suitable for a broader range of IOL types. However, most transscleral-sutured methods are time-consuming and traumatic because of the need to create scleral flap(s)/pocket(s)/groove(s). In addition, concerns about the long-term stability of the conventionally used 10-0 polypropylene are growing due to the risk of suture breakage or degradation over time [6–10].

To fixate the suture to the scleral wall with minimal invasiveness and to reduce suture-related long-term instability, we proposed a conjunctiva-sparing transscleral suture fixation technique [5]. The present technique has several advantages over traditional transscleral-sutured fixation. First, it simplifies surgical procedures with minimal invasiveness, enabling the introduction of a suture loop, securing the suture to the haptics, and burying the suture free ends and knots without the need for creating flaps [10, 18]. Second, the 8-0 polypropylene used in this technique has greater durability and fatigue resistance than the conventional 10-0 polypropylene, which is promising for establishing long-term stability between the suture and the haptics and preventing suture breakage. Third, the modified cow-hitch knot used to anchor the suture to the haptics is a non-free-end fixation technique. The incarceration ability of the fixation technique without a free end is secured by the friction between the suture and the haptic. The friction of different types of lasso techniques used to fixate IOLs is mainly determined by two factors: the contact areas between the suture and the haptic; and the overlapping areas of the suture. Compared with the conventional 10-0 polypropylene suture, the 8-0 polypropylene used in this technique has a wider diameter, providing more contact areas to enhance the knot's friction and reduce its loosening and slippage.

The postoperative position of implanted IOLs varies with intraocular conditions and surgical techniques [19, 20]. Previous studies have confirmed that a certain degree of IOL tilt occurs even in patients undergoing routine cataract surgery, which is generally well tolerated [19, 21]. Others reported that the mean tilt angle after an in-the-bag IOL implantation ranged from 1.5° to 4.8° [19, 22, 23]. A transscleral-sutured IOL is expected to have a greater tilt angle due to the lack of capsular support. In a study comparing IOL position between conventional scleral suture fixation and primary in-the-bag implantation, Hayashi et al. found that the IOL fixated to the scleral wall exhibited a more pronounced mean tilt degree (scleral-sutured: 6.35° vs. in-the-bag: 3.18°) [24]. However, recent advancements in IOL fixation techniques in the absence of capsular support have led to a decrease in the degree of IOL tilt. Yamane et al. reported a mean IOL tilt degree of 3.4° after flanged IOL fixation [3]. Kumar et al. found that the tilt angle after glued fixation was 3.2° and 2.9° in horizontal and vertical axes, respectively [8]. More recently, the study using a novel scleral anchored IOL revealed that the tilt angle was approximately 1° [25]. The mean tilt angle was 2.33° ± 1.36° in our series, which is lower than 7°, indicating a nonsignificant tilt and is comparable to that of routine cataract surgery. Decentration is another critical parameter influencing long-term visual outcomes. It has been confirmed that patients with clinically significant decentration (decentration > 0.4 mm) would experience a worse visual quality than those without it [12]. Previous findings have demonstrated that IOL within the capsular bag exhibited less decentration amount than conventional transscleral-sutured one (in-the-bag: 0.29 mm vs. transscleral-sutured: 0.62 mm) [24]. Others showed that the range of postoperative IOL decentration following transscleral-sutured fixation was 0.31  to 0.45 mm [7, 26]. The introduction of intrascleral sutureless fixation has simplified the surgical procedures and reduced the potential risks of suture-related issues; however, reportedly, the decentration amount of IOL fixated via intrascleral sutureless approaches was approximately 0.35  to 0.42 mm, which was still larger than that of routine cataract surgery [19, 26–28]. In our study, the mean decentration was 0.21 ± 0.15 mm, consistent with routine cataract surgery [19, 28]. Possible explanations for the different extents of decentration between the intrascleral and the present technique were as follows: first, intrascleral sutureless methods require inserting the haptics into scleral tunnels, which are not securely fixated, and the haptics might slide before reaching its final position. In contrast, the present technique was a definitive knotting approach to secure the haptics to the scleral wall. Second, after sclerotomy, an angled intrascleral pass of the needle to create an intrascleral tunnel is needed to embrace the leading and trailing haptics in flanged fixation; therefore, there is variability in the location and/or length of the intrascleral tunnel compared to the in situ knotting fixation in the present technique.

Regarding IOL power calculation accuracy, all three formulas produced myopic errors, ranging from −0.29  to −0.50 D, in agreement with previous transscleral-sutured results, as the IOLs were implanted outside the capsular bag [24, 29]. The principle of the three formulas might account for the results because these formulas were introduced to predict IOL power within the capsular bag; however, the fixation sites in the present technique were 2.0 mm posterior to the limbus, anterior to the intracapsular implantation position, leading to myopic errors. The reported MAE following intrascleral or transscleral fixation using the third-generation formulas was 0.61 to 0.86 D, which agreed with our results [30, 31]; whereas the values of MAE in our series were larger than those of routine cataract surgery, suggesting the compromised performance of formulas when the IOL was placed outside the capsular bag [32]. Previous studies reported that 30% to 45% of eyes with a PE within ±0.5 D in patients receiving flanged or transscleral-sutured fixation, in accordance with our study (43.48%) [30, 33]. Furthermore, for cases following flanged or transscleral-sutured fixation, the percentage of a PE within ±1.0 D was approximately 60% to 75% [30, 33]. The U.K. National Health Service guidelines recommended that at least 85% should have a PE within ±1.0 D [34]. In the current study, nearly 85% of eyes had a PE within ±1.0 D using the Hoffer Q formula, indicating that the present technique can offer satisfactory calculation accuracy even in such complicated surgeries. However, given that nearly 95% of cases can achieve a PE within ±1.0 D in routine cataract surgery, large sample studies are needed to investigate contributors to calculation errors in patients receiving the present technique [32].

The present technique can safely manage eyes with inadequate capsular support. There were no intraoperative complications besides a mild and temporary hemorrhage. The postoperative complications were transient IOP abnormalities, IOL pupillary capture, and suture exposure. Transient IOP abnormalities were noted in 8 cases. They were successfully managed with topical medications except for one case who was diagnosed with traumatic angle recession glaucoma and received antihypertensive surgery according to the preoperatively determined surgical plan-fixating an IOL first and implanting a glaucoma drainage device afterward. Previous studies reported that IOL pupillary capture and suture exposure rates after scleral fixation were 2% to 8% and 2.5% to 10%, respectively [3, 35–37]. In our study, two cases of IOL pupillary capture and one case of suture exposure were observed, and the incidence rates were within the abovementioned ranges. Previous studies with a mean follow-up of 6 to 7 months reported that the rate of IOL subluxation/dislocation after transscleral-sutured fixation was 3.0% to 7.8% [38, 39]. Also, due to suture degradation, IOL redislocation with a long-term incidence rate of 18% to 28% remains a major concern after transscleral-sutured fixation [9]. Though it was not observed in the present study, a longer follow-up is needed.

We also acknowledged the limitations. This is a single-arm clinical trial whose IOL position and IOL power calculation accuracy were compared with those of previously published studies. Moreover, the sample size of this study is small, and the follow-up period for some cases is relatively short. Nevertheless, the present technique provides a reliable alternative to manage refractive errors in eyes without adequate capsular support.

## Figures and Tables

**Table 1:** Demographics of included patients.

Parameters	Values
Age (years)	57.39 *±* 14.83 (range: 26–79)
Male/female (*n*)	31/9
OD/OS (*n*)	19/22
Follow-up period (month)	7.46 ± 6.42 (range: 1–24)
Axial length (mm)	25.73 ± 2.88 (range: 22.46–32.20)
Indications (*n*)	
Aphakia	14
Subluxated lens	3
Intraocular lens dislocation	24

**Table 2:** Comparison of preoperative and postoperative clinical outcomes.

Parameters	Preop	Postop	*P* value
IOP (mmHg)	14.74 ± 3.59	14.40 ± 4.95	0.82
UCVA (LogMAR)	1.28 ± 0.74	0.58 ± 0.40	<0.001
BCVA (LogMAR)	0.52 ± 0.62	0.31 ± 0.36	0.01
SE (D)	4.50 ± 6.38	−1.68 ± 1.57	<0.001

BCVA = best corrected visual acuity; D = diopter; IOP = intraocular pressure; LogMAR = logarithm of minimal angle of resolution; preop = preoperative; postop = postoperative; SE = spherical equivalent.

**Table 3:** IOL tilt angle and decentration amount.

Meridian	Tilt (°)	Decentration (mm)
Vertical axis	2.90 ± 1.93 (range: 0.39–9.10)	0.23 ± 0.19 (range: 0.02–0.94)

Horizontal axis	1.75 ± 1.41 (range: 0.24–7.65)	0.18 ± 0.19 (range: 0.02–1.06)

Mean	2.33 ± 1.36 (range: 0.51–6.17)	0.21 ± 0.15 (range: 0.02–0.64)

**Table 4:** IOL power calculation accuracy of 3 IOL formulas.

Formulas	Mean PE ± SD (D)	MAE ± SD (D)	MedAE (D)	Within ±0.5 D (%)	Within ±1.0 D (%)	Within ±2.0 D (%)
SRK-T	−0.50 ± 0.97	0.81 ± 0.72	0.55	43.48	69.57	95.65
Holladay 1	−0.36 ± 0.97	0.76 ± 0.68	0.70	43.48	73.91	95.65
Hoffer Q	−0.29 ± 0.93	0.70 ± 0.66	0.74	43.48	82.61	95.65

D = diopter; MAE = mean absolute error; MedAE = median absolute error; PE = prediction error; SD = standard deviation.

## Data Availability

The data analyzed in this study are available from the corresponding author upon reasonable request.

## Supplementary Materials

Supplementary Materials Supplementary 1: Video 1: Animation of making the modified cow-hitch knot on the haptics and fixating the haptics to the scleral wall using the flapless intrascleral knotting technique. Supplementary 2: Video 2: Surgical record of a case with aphakia achieving IOL fixation with the aid of the modified cow-hitch knot and the flapless intrascleral knotting technique.
