# Supplementary material for: Flapless and Conjunctiva-Sparing Technique for Transscleral Fixation of Intraocular Lens to Correct Refractive Errors in Eyes without Adequate Capsular Support
Source: J Ophthalmol. 2023 Apr 19;2023:4032011. doi: 10.1155/2023/4032011 (PMC10132900; doi:10.1155/2023/4032011)
Supplement: Supplementary Materials — Supplementary 1: Video 1: Animation of making the modified cow-hitch knot on the haptics and fixating the haptics to the scleral wall using the flapless intrascleral knotting technique. Supplementary 2: Video 2: Surgical record of a case with aphakia achieving IOL fixation with the aid of the modified cow-hitch knot and the flapless intrascleral knotting technique. [file 4032011.f1.docx]

Supplementary videos can be downloads from:

https://1drv.ms/u/s!AuJrN7HKiFozlXojdAbdjdSY4Kq0?e=0f2e9B
